# Creating a healthy and sustainable food environment to promote plant-based food consumption: clear barriers and a gradual transition

**DOI:** 10.1186/s12889-024-19121-5

**Published:** 2024-06-17

**Authors:** Ward S. van Hoeven, Monique Simons, Melina T. Czymoniewicz-Klippel, Harm Veling

**Affiliations:** 1 Consumption and Healthy Lifestyles, Wageningen University & Research, P.O. Box 8130, Hollandseweg 1, 6700 EW Wageningen, The Netherlands; 2 Behavioural Science Institute, Radboud University, P.O. Box 9104, Thomas Van Aquinostraat 4, 6525 GD Nijmegen, The Netherlands

**Keywords:** Healthy and sustainable purchases, Food environment, Protein transition, Intervention implementation

## Abstract

**Background:**

A shift away from diets high in animal-based foods towards diets high in plant-based foods is desirable considering human health, environmental sustainability, and animal welfare. As the food environment plays a crucial role in shaping consumption patterns, understanding of how changes in the food environment can facilitate plant-based consumption is crucial for the so-called *protein transition*. The current study aims to garner insight into barriers and facilitators for food outlet managers to take action to stimulate plant-based consumption within a local food environment.

**Methods:**

Using a maximum-variation sample approach, we examined possible barriers and facilitators to promote plant-based consumption across different types of food outlets located within a geographically shared food environment (a city in the Netherlands). We conducted in-depth semi-structured interviews among food outlet managers and applied multi-stage thematic analysis to the interview transcripts.

**Results:**

Most managers underscored the urgency of shifting towards more plant-based diets, and perceived a growing demand for plant-based products. However, three barriers hindered most of them from taking decisive action: Managers’ perception of low consumer demand for plant-based food options; fear of consumer resistance when stimulating plant-based food options; and limited behavioral agency to offer attractive plant-based food options. The few managers who made changes, or intend to make changes, are individuals with high intrinsic motivation, knowledge and skills.

**Conclusions:**

The present work suggests the key for change towards a food environment stimulating plant-based consumption lies in addressing three (perceived) barriers shared among diverse outlets. These are partly different from barriers for stimulating healthy consumption in general. Furthermore, current changes appear to be driven incidentally by individuals who are motivated and able to stimulate more plant-based purchases among a small targeted group of consumers.

**Supplementary Information:**

The online version contains supplementary material available at 10.1186/s12889-024-19121-5.

## Background

### The need for a dietary shift

Western diets containing a relatively high amount of meat and other animal-based foods and a relatively low amount of plant-based foods are related to multiple urgent health and sustainability issues. First, diets high in animal-based foods, and especially red and processed meat, rich in saturated fatty aid, are related to obesity (compared to diets lower in red and processed meat; [1]). Omnivore diets are related to increased mortality rates and non-communicable diseases such as hypertension compared to vegetarian, and especially vegan diets (Orlich et al., [2, 3]). Lower health risks (e.g., diabetes 2 and coronary heart disease) are associated with diets relatively high in plant-based foods, and in particular with diets high in healthy plant-based foods such as whole grains, fruits, vegetables, nuts and legumes [4, 5]. Such healthy plant-based diets offer a protective cardiometabolic advantage compared to omnivorous diets that are also considered healthy, i.e., containing the recommended amount of vegetables, fruits and grains [6]. Second, compared to diets relatively high in plant-based foods, diets high in animal-based foods, specifically red meat and dairy, have a higher environmental impact, i.e. more greenhouse gas emissions, land use, energy use and acidification- and eutrophication potential [7, 8]. Third, intensive livestock farming, as is common in Western countries, results in poor animal welfare [9, 10].

Consequently, there is increasing attention to moving away from animal-based foods as the main source of foods in diets, towards more plant-based foods. This shift is commonly referred to as the “protein transition”. The protein transition is not only endorsed by science (see e.g., [8]), but also increasingly translated to practice via, *inter alia*, dietary guidelines for the general public (for example, by the Netherlands Nutrition Centre, see [11]). Still, consumption of meat per capita is rising worldwide, is stable in Europe [12] and is barely declining in the Netherlands [13]. This raises the question what makes the protein transition so difficult.

### The potential role of local food outlets

**Fig. 1:**
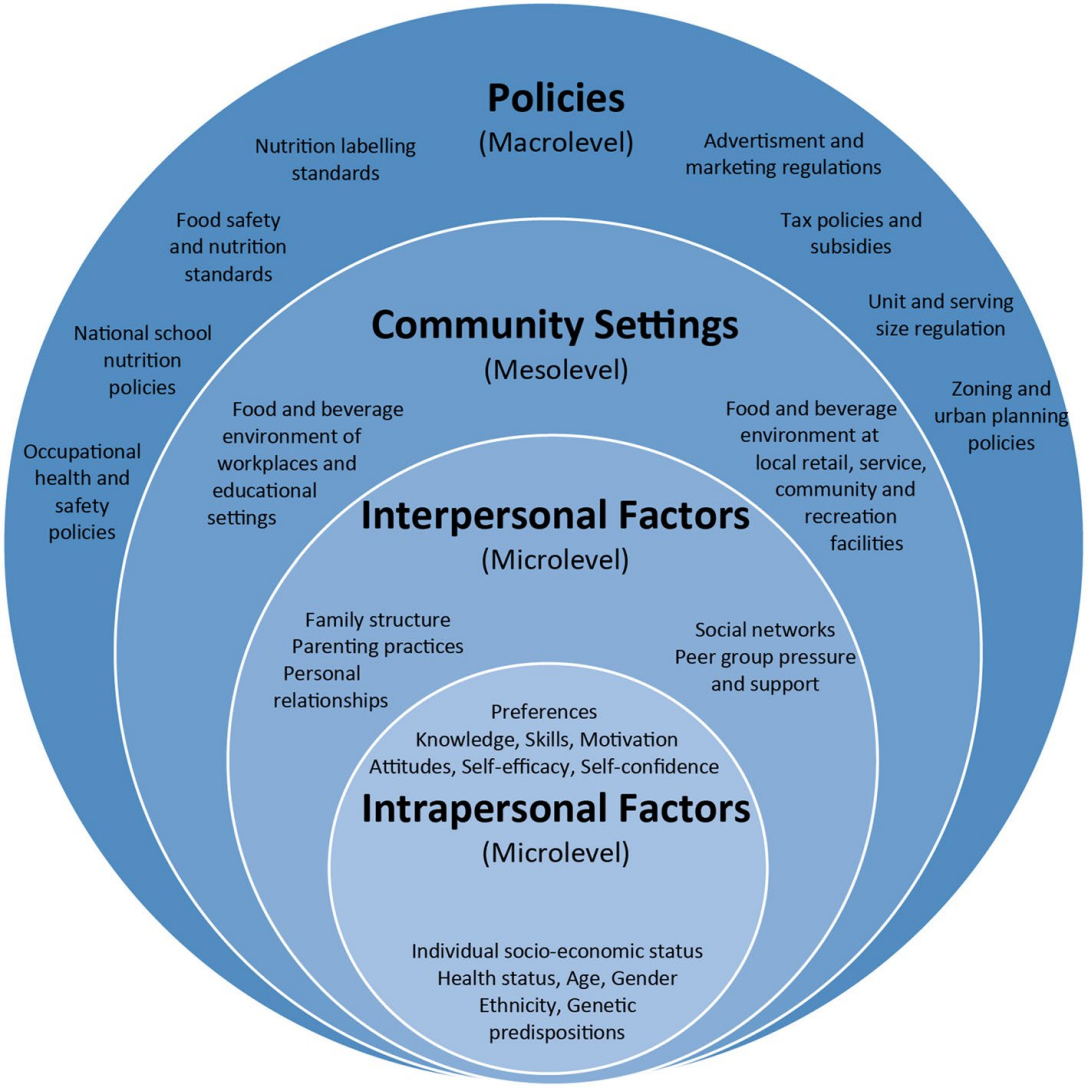
Socio-ecological model on food and beverage intake, reproduced with permission from von Philipsborn et al. [15]. The figure shows food consumption is shaped by factors on different levels such as biases in individual decision making (intrapersonal level), parental practices (interpersonal factors), the food environment (community settings) and taxes (policies)

One way to understand difficulties in shifting dietary patterns, and identify possible ways to intervene, is by approaching food consumption from the socio-ecological model (see Fig. 1). The socio-ecological model places dietary consumption in a multi-layered system, suggesting different possible leverage points for dietary transition (see e.g., [14, 15]).

The socio-ecological model makes clear how realizing the protein transition is a complex process, involving many different factors and stakeholders dynamically influencing each other over time [16, 17]. Therefore it has been suggested that for the protein transition, interventions should target multiple levels in the system in parallel [18]. While acknowledging this complexity, in the current study, we zoom in on the community level, investigating the possible role of voluntary action by food outlets to stimulate more plant-based consumption by making changes in the food environment. Broadly defined, the food environment is the interface between consumers and the food system, and is defined by the availability, affordability, convenience, and desirability of various foods [14]. In our focus on the role of food outlets, we operationalize the food environment by focusing on available products in food outlets (availability), price of these products (affordability), positioning of the products (convenience), and promotion of these products (connecting to desirability of various foods). By using the 4P marketing mix of Product, Price, Promotion, and Position (e.g. see [19]), we consider the elements of the food environment that food outlet managers may modify. Food outlets collectively shape the food environment and influence consumers in their purchases by making available various food options and marketing a selection of foods [20–22]. Consumers are thus affected by not only one, but by a diversity of outlets, such as supermarkets, restaurants, snack bars, canteens, cafés and butchers, in the geographical area in which they purchase their food. Therefore, efforts to intervene in the food environment should consider the different types of outlets and their willingness and ability to take action.

Managers of outlets may be influenced by neighboring outlets. For instance, Ghosh-Dastidar et al. [23] suggested an increase in healthy food offerings in one outlet may stimulate neighboring outlets to segment towards unhealthier foods. Such a negative spillover may counteract actions by outlets that are willing to make changes to stimulate more plant-based purchases. Conversely, a spillover might also be inducive for the protein transition if the actions of one outlet would set an example for others to do the same. Currently, there is no literature on how outlets perceive and react to outlets around them stimulating plant-based versus animal-based purchases.

Only few studies about the willingness and ability of food outlets to stimulate more plant-based purchases specifically have been conducted [19, 24–27]. To understand how the food environment can be steered towards stimulating more plant-based purchases, we need a better understanding of what drives food outlet managers. In the present research, we examine the barriers and facilitators for stimulating more plant-based purchases among managers of a variety of food outlets to gain insights into what role they could have in facilitating the protein transition.

Relative to research on stimulating plant-based purchases specifically, there has been more attention to stimulating healthy choices generally, which often includes stimulation of plant-based foods, but also healthy animal-based foods. Despite overlap between healthy and plant-based foods, the motivation to eat healthy may be of a different nature than the motivation to also eat sustainably and animal-friendly [28], which may lead to different barriers and facilitators for changing the food environment towards healthy versus more plant-based. Furthermore, the availability of products or skills to prepare dishes may be different for healthy compared to plant-based food options.

### Barriers and facilitators for outlets to stimulate healthy purchases

Based on 25 reviews, Gupta et al. [29] collated a list of factors that affect the implementation of healthy food interventions in food stores, cafés, restaurants and vending machines. The review shows that *individual level factors*, such as lack of retailer knowledge and skills and low perceived demand for healthy foods are found to be key barriers for implementing healthy food retail interventions (such as offering healthy food by default or placing healthy foods in sight). Managers’ openness to innovation and feelings of responsibility for community health were facilitators. At the *interpersonal level*, by engaging consumers and considering their preferences and demands, retailers are reported to be better able to implement interventions. Furthermore, establishing a relationship between retailers and intervention developers is suggested as vital for ensuring that the proposed intervention fits the context of the retailer. *Environmental level factors* found by the review included profitability, autonomy to implement changes (possibly limited by, for example, contract agreements) and available resources, such as staff, time, capital and physical in-store space. Furthermore, policies, such as a governmental healthy food assistance program [30], may enable the implementation of healthy interventions by increasing consumer demand for healthy food products.

### Barriers and facilitators for outlets to stimulate more plant-based purchases

For healthy food retail interventions that specifically stimulate plant-based purchases only a handful of studies [19, 24–27] have been published, and these have focused on only two types of outlets: Supermarkets and restaurants.

With regards to supermarkets, there have been studies investigating the barriers and facilitators on intervening in their outlet to stimulate consumers to shift towards relatively more plant-based purchases [19, 25]. In these studies, intervening related to making marketing-mix changes, such as portion size caps for meat products, increasing plant-based product saliency, attractive labelling of plant-based products, or price promotions. Trewern et al. [19] found that UK supermarkets are willing to offer more vegetarian and vegan purchases, but unwilling to disincentivize animal-based products, as they are afraid the latter might create resistance among consumers. This anticipated resistance is reported less as a barrier in studies about stimulating more healthy, and less unhealthy purchases [29]. Brimblecombe et al. [31] do report that while some managers of food stores initially expected consumer backlash towards healthy food store interventions, this did not occur. Furthermore, similar to studies about healthy food retail interventions [29], Trewern et al. [19] suggest that the main driver that shapes supermarkets’ practices around stimulating meat versus vegetarian purchases is the perception of what consumers want. This demand-driven strategy is reported to be shaped by supermarkets’ business model, which is geared towards increasing revenue [19]. Similar to the perception of high demand for unhealthy foods [29], supermarkets also have this perception for meat and dairy [19]. Therefore, they see it as a difficult topic to take the lead on, as it would cause a competitive disadvantage. Indeed, among different strategies to stimulate vegetarian and vegan purchases, pricing strategies, such as discounts, were found to be the least implemented in supermarkets [19], despite evidence suggesting these would be most effective [32]. Notably, this contrasts with promoting healthy food, as food outlets, including supermarkets, are willing to implement pricing strategies to stimulate healthy purchases [29].

Gravely and Fraser [25] suggest that, as supermarkets are risk-averse in “pushing” new plant-based products, their unwillingness to stimulate plant-based options will likely impede the success, i.e., high product turnover, of those products and are enforcing supermarkets’ perception of insufficient demand. This self-fulfilling prophecy of low demand for plant-based options, caused by the fear of revenue loss, is suggested to maintain supermarkets’ unwillingness to take strong action.

Other studies [24, 26, 27] have investigated barriers and facilitators for independent restaurant owners to stimulate plant-based purchases. Findings suggest that vegan restaurants emerge not primarily from an entrepreneurial-, but from an activistic drive [24, 27]. These studies suggest that owners of such restaurants are driven to run a plant-based restaurant to further express their existing vegan or vegetarian identity and because they want to promote health in their community. While some food stores also reported being motivated to implement healthy food retail interventions for community health [33], this seems less pivotal than the drive regarding stimulating plant-based diets.

Some restaurant owners believe demand for plant-based food is low due to consumers having negative pre-conceptions about flavor and satiation of vegetarian and vegan meals [27]. Moreover, Crimarco et al. [24] found that creating attractive vegan options is perceived challenging. Finally, Rivera & Shani [26] studied barriers for offering vegetarian and vegan dishes among restaurants that are not mainly plant-based, and found perceived profitability of those dishes to be mentioned as a barrier to include them.

### The present study

The present study builds on existing studies by employing a maximum variation sampling approach to examine perceived barriers and facilitators to stimulate more plant-based and fewer animal-based purchases among a variety of outlet owners located in the same city. The advantage of this approach is its ability to examine similarities and differences within a heterogeneous group [34]. Moreover, consumers are influenced by the combination of food outlets in an area, which together constitute the food environment. Diets may not change much when only one type of outlet changes and the other outlets remain unchanged. Therefore, we will examine multiple types of outlets in one geographical area. This also allows us to examine how food outlets may perceive and react to nearby outlets located in the same area. Altogether, in exploring the potential for managers of different types of food outlets to stimulate plant-based purchases, the current study will answer the following questions:

1. What are food outlets in a city in the Netherlands currently doing, and planning to do, to stimulate more plant-based and fewer animal-based purchases?
2. Which barriers and facilitators affect what these outlets are doing, and planning to do, to stimulate more plant-based and fewer animal-based purchases?
3. How do managers of food outlets think they are influenced by the food outlets around them with regards to stimulating more plant-based and fewer animal-based purchases?

## Methods

### Design

#### Participants

Managers of outlets within the city were selected for maximum variation of outlet types, and contacted through an employee of the local municipality office if they were part of their network (*n* = 7), or directly by the first author by telephone or email (*n* = 18). Within the maximum variation strategy, convenience sampling was used, i.e., for each type of outlet, one participant was recruited through convenience sampling. Out of the 25 invited food outlet managers, 12 participated, 6 rejected; 5 did not reply; 2 accepted but failed to follow-up. Reasons for not participating were time restrictions, mostly due to a staff shortage (*n* = 4); the belief that a local outlet cannot change anything (*n* = 1); and distrust in the correct representation of interview data (*n* = 1). The final sample includes: supermarkets (franchise, branch and independent organic); restaurants (eat-in and take-away); a butcher; a snack bar; a food bank; canteens (at a large organisation and at a sports association); and a lunch café (see Table 1). The study took place in the Netherlands, in which meat reduction is a topical issue [35]. Dutch consumers have a growing intention to eat less meat (Natuur & Milieu [36]), yet meat consumption per capita is barely declining [13]. Data [37] show that a small group of the adult Dutch population identifies as vegetarian (2,1%) or vegan (0,4%), while 20,2% of people report eating meat every day. Besides these strict vegetarians, vegans and meat eaters, the largest proportion of people report eating meat some days of the week (44,7% 1–4 days/week; 30,4% 5–6 days/week). The same data show that 35% of people state having decreased their meat consumption in the past year, while 37% of people that consume meat state that they should eat less meat. The city in which the outlets in the sample are located is a medium-large (100.000–200.000 inhabitants) Dutch city.

**Table 1:** Outlet type and respondent role per interview

Respondent	Outlet type	Role
R1	Tapas restaurant	R1: Co-owner and co-manager; responsible for the general management of the restaurant
R2	Butcher	R2: Co-owner, co-manager and salesperson; responsible for preparing products
R3	Snack bar	R3: Manager; responsible for new product development, product assortment and procurement at suppliers
R4	Franchise supermarket	R4: Assistant store-manager; responsible for ensuring a well-stocked store and co-responsible for ensuring revenue
R5	Branch (affiliate) supermarket	R5: Operations-manager; responsible for ensuring a well-stocked store and co-responsible for ensuring revenue
R6	Food bank (charitable food aid organization)	R6: Main manager; responsible for the general management of the food bank
R7	All-you-can-eat and take-away Chinese restaurant	R7: Owner, manager and host; responsible for the menu, procurement at suppliers
R8	Sports canteen	R8a: Chair of the bar-committee; responsible for the menu and for recruiting volunteers for behind the bar R8b: No official role; helping to create dishes and volunteer in the kitchen
R9	Municipality canteen (from the perspective of the municipality)	R9a: Strategic advisor facility management; responsible for catering and hospitality R9b: Assists R9a in their tasks
R10	Caterer (external company, active in municipality canteen)	R12: Regional manager; responsible for managing on-sight canteen managers
R11	Café	R11: Co-owner and co-manager; responsible for the general management of the café and partly for preparing dishes
R12	Organic, package-free store, run as a cooperation	R12a: Co-partner; responsible for managing the store R12b: Co-partner; responsible for managing the store

#### Materials

The interview guide was based on the literature review. It consisted of the following themes: Interviewees’ current and prospective practice around plant-based options versus animal-based options; the factors that influence their current and prospective practice; their ability to implement changes; and the effect of changes at other outlets on their own practice.

The interview protocol considers that managers may perceive changing certain elements in their outlet, to stimulate plant-based purchases, more feasible and desirable than changing other elements. Drawing on earlier research [19, 38], our study investigates the modifiable elements within food outlets, employing the 4P marketing mix: Product, Price, Promotion, and Position. The application of the 4Ps has demonstrated its utility in identifying potential intervention strategies, as observed in a comparable study which looked into reducing animal-based purchases in supermarkets [19]. Product refers to the offer of vegetarian, vegan and meat options and the ratio between those. Stimulating more plant-based, and fewer animal-based, purchases may entail different things for different outlets. For outlets that currently have few vegetarian options, going “more plant-based” might entail doing more with vegetarian options. For outlets that already have many vegetarian options, moving towards more plant-based might entail doing more with vegan options. Therefore, we will sometimes refer to both vegetarian and vegan options as “more plant-based”, depending on this context.

Within product, we additionally consider the portion size of animal-based ingredients, as reducing the amount of animal-based ingredients in animal-based options will also lead to lower animal-based foods consumption. Price focuses on the difference between vegetarian, vegan and meat options. Intervening on price, for instance, by increasing the relative price of meat, is suggested to be an effective intervention [32]. Promotion entails product deals, but also, for example, verbal suggestions made while interacting with customers. Position refers to the location of products in an outlet, as well as positioning of different options on the menu. Positioning more plant-based options and animal-based options together could facilitate “product swaps”, which has been suggested as promising in stimulating supermarket customers to choose healthier products [32]. Using the 4Ps as a framework helps to identify which food environment elements may have potential to be changed from the perspective of food outlet managers.

Furthermore, we distinguish three types of replacements for meat and other animal-based foods: Vegetarian options, vegan analogue replacements and vegan natural replacements, such as low-processed nuts, beans and legumes. Outlets may have a different perspective on vegetarian versus fully vegan options. Also, while vegetarian and vegan analogue replacements may have an important role in the protein transition as they offer a convenient alternative to meat for consumers, vegan natural replacements such as legumes are superior with regards to health and sustainability [39]. Therefore, the perspectives from outlets on all three categories are of interest.

The first author conducted the interviews and started with introducing the research and the research aims and explaining the interview structure. See the appendix for the full interview protocol.

#### Procedure

Data were collected between October and December 2022 by the first author. Consent was obtained from all participants prior to the interview. Ethical committee approval was attained at Radboud University Ethics Committee of the Social Sciences Faculty (reference: ECSW-LT-2022–10-18–9087).

The interviews (*n* = 12) were executed by video-call (*n* = 9) or, when participants requested that, in-person (*n* = 2) or over the phone (*n* = 1) and lasted on average 45 min. The interviews were audio recorded and transcribed verbatim, or if the interviewee did not allow that (*n* = 1), detailed minutes were made by a research assistant during the interview.

### Analysis

The interview transcripts were analysed in ATLAS.ti using multi-stage thematic analysis, using a mix of deductive and inductive coding [40, 41]. Based on the interview protocol, an initial set of codes was formed. This consisted of, for each of the 4Ps: Outlets’ current and prospective practice; the factors that influence their practice; outlets’ autonomy to make changes; requisites for implementing changes; and outlets’ perceptions and reactions to changes in other outlets. In a first round of coding, the data was coded with these deductive, as well as with new inductive codes. Subsequently, the data segments within each code were inductively recoded into more specific sub-codes (*n* = 212) and then clustered into themes. Within each theme, codes were marked as barriers or facilitators for outlets to stimulate plant-based purchases. For the final analysis, themes were compared between the 4Ps and between outlets.

One-third of the interviews were coded by a second coder (ML) to reduce coder bias. Small discrepancies, about which deductive code a text segment fits best to, were discussed between the first (WH) and second coder. In the few cases there was no clear consensus between the two coders, a third researcher (MS) was consulted to reach consensus.

## Results

In Sect. “Current and prospective practices’’, outlets' practices around stimulating more plant-based and less animal-based purchases are described for each of the 4Ps. Next, the underlying barriers and facilitators for those current and prospective practices are laid out.

### Current and prospective practices

An overview of current interventions to reduce meat or promote plant-based purchases that were reported is presented in Table 2. Vegetarian options were offered in all but one (R8: sports canteen) outlet. Meat was predominant over plant-based options in all but three outlets (R1: tapas restaurant, R9&10: municipality canteen, R12: organic store). The municipality canteen (R9&10) served vegetarian food by default when employees order a catered work lunch, while still offering the possibility to opt for meat. Only the municipality canteen used reduced portion size of animal-based ingredients in dishes.

**Table 2:** Current interventions to reduce meat or promote plant-based purchases

Element	Respondent	Example
Product	R1 (tapas restaurant), R9&10 (municipality canteen), R12 (organic store)	Decrease portion size of animal-based products (R12: organic store)
Place	R1 (tapas restaurant), R7 (Chinese restaurant), R12 (organic store)	Place animal- and plant-based options mixed on the menu (R1: tapas restaurant, R7: Chinese restaurant)
Promotion	R1 (tapas restaurant), R12 (organic store)	Suggest vegetarian option when customers ask for meat (R12: organic store)
Price	R4 (franchise supermarket), R5 (branch supermarket)	Plant-based options on offer (yet less so than animal-based options)

The price of animal-based versus vegetarian, versus vegan options were reported to be comparable by all respondents, with the exception of the organic store (R12) that only offered relatively expensive organic meat, i.e. this was not a deliberate pricing strategy.

The municipality canteen (R9&10) promoted more plant-based options verbally by suggesting a vegetarian option when customers ask for meat. Another retailer (R1: tapas restaurant) promoted vegan options through online promotion on social media and by telling customers in their restaurant that the dish they ate, for example a cheesecake, was vegan, in case the customers did not see that on the menu. Products being on offer is a combination of price and promotion. In the supermarkets (R4, R5), more plant-based products were reported to be on offer regularly, but much less frequently and less saliently, i.e. smaller shelf-facings, compared to meat (R4).

The positioning of plant- versus animal-based options varied between outlets. Some restaurants deliberately put the different options mixed on their menu (R1: tapas restaurant, R7: Chinese restaurant), while all grocery stores (R4, R5, R12) had them in separate physical sections. Interestingly, some outlets did not put all vegan dishes or sandwiches on their menu (R7: Chinese restaurant, R11: café) or did not put the vegetarian hamburgers in the counter (R2: butcher), meaning clients would need to ask for additional vegetarian or vegan options, which exist but are not visible. In contrast, the municipality canteen (R9&10) intentionally positioned plant-based options more prominently by putting some of the meat options behind the self-service counter. Conversely, the food bank (R6) positioned vegetarian options after the meat options, and such that these were presented as an extra instead of a substitute for meat.

Many retailers stated that in the future they would like to move towards a more plant-based product offer and that they could see themselves doing more with promoting and favorably positioning more plant-based options. However, structural pricing strategies were only an option for one outlet (R1: tapas restaurant) and reduced portion sizes were an option for two respondents (R1: tapas restaurant, R10: municipality canteen caterer). In sum, it appears that incidentally there is some stimulation of plant-based purchases, yet focus remains mostly on animal-based options.

### Barriers for outlets to stimulate more plant-based purchases

#### Perceived demand

Even though all respondents, except from the food bank (R6), stated they would like to see people eat less meat, many reported it would not be viable to remove (a part of) their meat options. The bottom line for all outlets, except for the food bank, was creating enough revenue. Therefore, perception of demand for animal- and plant-based options seemed to be the foremost factor, or at least a crucial prerequisite, behind the offer for managers. Although perspectives varied, most participants spoke of a risk of revenue loss when making the offer more plant-based. Demand for meat and dairy was perceived as high by all interviewees but one (R1: tapas restaurant).

Perceived demand also played a role in offering plant-based options. Operating in a niche of vegan-minded consumers, one respondent (R1: tapas restaurant) perceived a large demand for more plant-based options. All respondents except for from the tapas restaurant and the municipality canteen (R9) and their caterer (R10) perceived a low demand. Some participants feared that a lack of demand for more plant-based options would result in too little turnover and thus spoilage. In some cases, the low perceived demand was based on sales data. Two retailers (R4: franchise supermarket, R7: Chinese restaurant) had unsuccessful attempts to stimulate purchases of more plant-based options by increasing the offer thereof (most of which did not sell enough); by using positioning (certain vegan products displayed more prominently; which had no substantial effect on sales); promotions (on social media; which had no perceived effect on sales); and reduced amounts of animal-based products in dishes (more vegetables and less meat; which resulted in complaints from customers). The manager of the Chinese restaurant explained, *“I tried doing more vegetables and less meat, but people noticed immediately. They don’t want that.”*

In other cases (R2: butcher, R8: sports canteen), perceived demand for more plant-based options was influenced by frequency of clients asking for those options, while not selling them nor having them visible in the outlet. In the franchise supermarket (R4), higher perceived demand for meat was reflected in how promotions were executed: meat promotions were expected to create more turnover and therefore get larger, i.e. more salient, shelf-facings.

Geographical outlet location was mentioned by two participants (R2: butcher, R7: Chinese restaurant) as a determinant of demand, as consumers in certain areas, such as working-class districts, were perceived as having very little demand for more plant-based options. The manager of the Chinese restaurant said: “*Personally, I want vegetarian. But at this location it’s not possible.”* Some participants also thought that consumers not yet being familiar with more plant-based options is a reason for low demand.

Food outlets mentioned hospitality towards consumers as an important consideration. They want everyone, including meat-eaters, to feel welcome and to not feel pushed towards any diet. Even outlets that would ideally have a fully vegan assortment (R9: municipality canteen) wanted to keep most, or in the case of the tapas restaurant (R1), at least some, of their animal-based options and to also keep those affordable (R9: municipality canteen). Especially for stores, and particularly supermarkets, respondents mentioned that the clientele group is highly heterogeneous and thus the offer should cater to many different needs.

*Some people simply want to eat meat. So that relates to also being a hospitality business. You want all of your customers to be happy. Eh, so for people who really want it, we can do that, we do that.”* – Municipality canteen (R9)

Some respondents reported negative beliefs regarding vegetarian and vegan products that hinder demand. For instance, some respondents attributed a large environmental impact to soy or perceived a risk of nutritional deficiencies when not consuming meat:

*“But then you need to be knowledgeable on nutrition. That you get enough nutrients […] the other day there was a customer who came back to the shop. He said he stopped eating meat for a while. But he said: I came back to that. And my muscles, my muscle-mass is growing again. He says he is feeling much better.” –* Butcher (R2)

#### Fear of resistance

Fear of resistance, or being paternalizing and losing customers, inhibited respondents to take strong action, especially regarding pricing and portion size interventions. Many respondents did not want to use promotions that they perceive as intrusive, or as preaching, to prevent resistance among customers. Therefore, as explained by the municipality canteen representative, only promotions that are gradual and careful were deemed feasible:

*“So, we are really only at the start yet. Now, it is about very gently, softly suggesting like: Look, there are some plant-based things, which you might like, would you care to try it once?”* –Municipality canteen (R9)

The food bank (R6) manager reported that their customers have other priorities i.e., food security, and said: *“I personally think [the protein transition] is very paternalizing. We think that.”* In the sports canteen (R8), resistance was also feared from within the organization. The respondents expressed fear about the volunteers behind the bar and in the kitchen not being willing to volunteer anymore if they have to also prepare more plant-based dishes, which would complicate their tasks, or if they would need to verbally promote more those, which some volunteers were believed to not personally endorse. Many respondents believed that awareness of consumers and acceptance of a more plant-based diet was not yet high enough. Some outlets reported seeing a role for themselves in the process of familiarization by exposing consumers to more plant-based options. Yet they believed that this should be a slow, gradual process, to prevent resistance.

#### Behavioral agency

Behavioral agency, that is, the ability to change the food outlet’s food options and marketing thereof as a manager, was a barrier in three ways. First, autonomy to make changes in the outlets was dependent on the type of outlet. Managers of outlets that were tied to a larger organization (i.e., R4: franchise supermarket, R5: branch supermarket, R9: municipality canteen), reported being limited in their autonomy to make changes. Within the branch supermarket, being part of a national chain, the manager stated having no direct influence on the product offer, price, and contents of promotions. The manager could theoretically ask the head-office to analyze, for example, the sales of vegetarian products in their outlet, to see if an expansion would be justified. The branch supermarket manager expected this would not turn out in favor of more plant-based products. The very limited autonomy the branch supermarket manager reported having is on changing the positioning of their offer and the positioning of, and the space allocated to promotions. For the franchise supermarket (R4) manager autonomy was reported to be similarly limited, although the decision to change the offering could be made locally. For the municipality canteen (R9) manager autonomy also was limited, as it is part of a larger political organization in which the employee council and political parties also have a say, yet did not all agree. Lack of agency could also take another form. One respondent (R7: Chinese restaurant) thought that they are too small to make a change, after multiple unsuccessful attempts to stimulate more plant-based purchases, and that science should make people aware of the benefits. Another respondent (R4: franchise supermarket) believed that self-regulation of businesses will not work, and suggested that more governmental intervention is needed.

Second, with regard to the availability of food options, some interviewees (R1: tapas restaurant, R3: snack bar) were positive towards (some of) the available vegetarian and vegan options from suppliers, whereas others (R2: butcher, R8: sports canteen, R11: café, R12: organic store) stated that they felt like there are no, or few, tasty and affordable options available. However, the respondents from the butcher and sports canteen had very little experience with tasting or working with vegetarian and vegan products. Barriers mentioned for reducing animal-based portion sizes were the unavailability of smaller packages of animal-based products as well as the taste of hybrid meat, i.e. a mix of meat and plant-based ingredients.

*“Because we are working with so many different parties. We, [name of R11] and me, might have an opinion, but we are a municipal organization, in which everyone wants to think along.”* – Municipality canteen (R9).

Third, for respondents from outlets that prepare food themselves agency was relevant in relation to the ability to prepare attractive plant-based options. Five respondents (R1: tapas restaurant, R3: snack bar, R7: Chinese restaurant, R10: municipality canteen caterer, R11: café) felt able preparing vegan options well, and one (café) reported enjoying developing this skill. Two others (R2: butcher, R8: sports canteen) considered cooking skills as a barrier for offering more plant-based options, although they thought they could learn these skills if they would invest time in it (see Table 3).

**Table 3:** Main barriers to reduce meat and increase plant-based options

Barrier	Respondent	Example quotes
Low perceived demand for plant-based; high perceived demand for animal-based	R2 (butcher), R3 (snack bar), R4 (franchise supermarket), R5 (branch supermarket), R6 (food bank), R7 (Chinese restaurant), R8 (sports canteen), R9 (municipality canteen), R10 (municipality canteen caterer), R11 (café), R12 (organic store)	*We would have substantially fewer customers if we would not sell any meat. I am a vegetarian myself. For me, this is a concession. –*Organic store (R12)
Fear of resistance or paternalization	R2 (butcher), R3 (snack bar), R5 (branch supermarket), R6 (food bank), R7 (Chinese restaurant), R8 (sports canteen), R9 (municipality canteen), R11 (café)	*The only thing is that I will make some people angry [by making the crispy fried chicken sausage plant-based]. But if it would only be about taste and texture, it would be easy. They will not even notice.* –Snack bar (R3)
Lack of behavioral agency (autonomy, availability, skills)	R2 (butcher), R3 (snack bar), R4 (franchise supermarket), R5 (branch supermarket), R8 (sports canteen), R9 (municipality canteen), R10 (municipality canteen caterer), R11 (café), R12 (organic store)	[a supermarket manager taking initiative to create a more vegetarian offer] *has a much lower chance of materializing than fifteen years ago, when there was still entrepreneurship in the stores*. –Branch supermarket (R5) *Sometimes, I make people a grilled cheese sandwich with vegan cheese. Or I should say: Vegan slices. Actually, it isn’t cheese, but eh, I don’t really like the taste […] And I know that there would be quite some visitors here that would have the same.* –Café (R11) *For me, cooking vegan is quite a challenge, I find it difficult. If I need to use, eh, eh, the juice from eh, chickpeas, to make eh, whipped cream, then that, that simply goes beyond my skills. I never looked into that and eh, I find it a difficult topic. And, eh, I think that if I don’t do that […] no one in the canteen will. –*Sports canteen (R8)

#### Other barriers

Participants also discussed practical barriers, such as needs to have space in the outlet for adding plant-based options, or that changing the offer or store layout takes time and money.

#### Perception of- and reaction to the practice of other outlets

While most respondents stated that they were not monitoring, nor being influenced much by surrounding outlets, two respondents (R2: butcher, R9: municipality canteen) stated that outlets around them posed a barrier for going more plant-based. The butcher believed they could not compete with the large assortment of cheap meat replacements from supermarkets. The municipality canteen feared losing canteen visitors to the many and diverse other food outlets close-by, if available meat options would be reduced or disincentivized through pricing or “aggressive” promotion.

Interestingly, some respondents did not consider stimulating vegan natural alternatives, i.e., legumes, nuts and seeds, as a strategy for reducing animal-based consumption in their outlet, even while in their personal life some of them reported doing so.

### Facilitators for outlets to stimulate more plant-based purchases

All but one respondent (R6: food bank) noted at least some urgency to reduce animal-based food consumption. Respondents reported they would like to see people eat more plant-based, because of environmental reasons (R1: tapas restaurant, R2: butcher, R4: franchise supermarket, R8: sports canteen, R11: café); animal wellbeing (R1: tapas restaurant, R7: Chinese restaurant) and health (R8: sports canteen). Another intrinsic motivator reported was the joy of experimenting with creating new, plant-based dishes (R3: snack bar, R11: café). Still, the data showed that these personal motivations did not always lead to action, as other factors such as low perceived demand and autonomy inhibited action.

While some outlets, and in particular supermarkets, seemed to be geared towards maximizing revenue over societal impact, other outlets reported more willingness to take action for the protein transition as long as it would not threaten the financial health of their company.

Most respondents perceived the protein transition as a development that will unfold in the coming 5–10 years, to become a product group that they need to have established. Therefore, they believed they should, in the near future, further develop their more plant-based range. As the butcher (R2) illustrated, many respondents whom perceived the demand for more plant-based options to still be too small to be economically interesting, did see the need for offering some vegetarian options to cater for groups that include people with those diets: *“For instance, when I have a barbeque for eh, thirty people, then often at least two or three will be vegetarian. And that, eh, that is of course going to increase.”* – Butcher (R2).

Similar to demand, the availability of tasty and affordable vegetarian and vegan options at suppliers was perceived differently among respondents. Multiple respondents reported that many animal-based products, such as cream, can be replaced one-on-one by plant-based analogues. According to the Tapas restaurant (R1) manager, *“you can easily replace things one-on-one, plant-based cream for animal-based cream, etcetera, etcetera.”* This contrasts the statement from a respondent from the sports canteen (R8), whom said that they would need to make whipped cream from the liquid from chickpeas, posing a barrier for them related to cooking skills.

While pricing strategies to stimulate more plant-based purchases were generally off-limits, in the organic store (R12) there was a financial incentive to buy less meat. The exclusively organic meat sold there simply was much more expensive than mainstream supermarket meat.

Although some respondents felt they have little power to influence purchases, others mentioned positive beliefs about the expected effect of implementing changes related to the 4Ps. Multiple respondents reported that an increase in their more plant-based offer, promoting with free tasters, or positioning more plant-based options in sight would likely lead to increased sales. For example, the snack bar (R3) manager said: *“Yes, part of it is that, when you start offering things, then, of course, they will be purchased.”* A practical argument mentioned by multiple respondents in favor of vegan is that it is convenient for catering to people with dairy intolerance or allergy.

## Discussion

Among a diverse sample of outlet mangers in a city in the Netherlands, there was high consensus that there is at least some urgency to move towards more plant-based diets. Interestingly, and despite this largely shared perception, current practices to promote plant-based over animal-based options were limited, and appeared incidental to the type of outlet and the individual who happens to be the manager. Relating to the first research question, i.e., what are food outlets currently doing and planning to do, almost all outlets involved in this study offered vegetarian options, and most also had vegan options, yet meat was predominant in all but one outlet. Positioning and promotion were used by some outlets to stimulate more plant-based and less animal-based options, and were also mentioned as strategies to be utilized more in the future (similar to [19]). Pricing strategies and reduced portion sizes of animal-based options were generally not being used by outlets to stimulate more plant-based and less animal-based purchases, and most managers reported being unwilling to start using these strategies. The discrepancy between privately held opinions and lack of substantial stimulation practices towards plant-based diets can be understood from the perceived or experienced barriers.

Despite the fact the sample consisted of managers of diverse outlets, three common barriers clearly emerged, relating to research question two: What barriers and facilitators affect food outlet managers? First, most outlet managers are hindered from taking action because of the concerns of losing customers, and thus revenue, when stimulating more plant-based and fewer animal-based purchases. Fear of losing customers when going more plant-based stems partly from the belief that there is currently little demand for such options, similar to prior research on plant-based [19, 25, 27] and healthy food interventions [29]. Indeed, some outlet managers encountered disappointing sales when attempting to stimulate more plant-based options. This aligns with recent previous work that shows consumers evaluate meat analogues more negatively than their meat counterparts (e.g., [42]). However, almost three-quarters of Dutch consumers indicate they want more plant-based products in outlets [43]. Indeed, other outlet managers in this study did successfully shift towards more plant-based. Altogether, these results raise the question to what degree, and in which cases, managers’ perception of low demand is correct.

There are two mechanisms that could explain how low perceived demand for plant-based options hinders the protein transition. First, low perceived demand will lead managers to not substantially “push” plant-based options, thus keeping the sales thereof low, enforcing this perception, as also suggested by Gravely & Fraser [25]. Second, disappointing plant-based sales may partly be due to lack of parallel action regarding dissuading animal-based options. In other words: if animal-based options remain as predominant and as attractively marketed as they are now, it might be difficult to persuade a substantial group of people to buy more plant-based. While almost all outlet managers reported seeing a trend towards more plant-based diets, and endorsed such a transition to least some degree, low perceived demand hampers change on the short term.

A second barrier to stimulate plant-based options via the food environment is the fear of possible customer resistance, or the risk of being perceived as paternalizing customers. Indeed, some outlet managers encountered resistance to change, which algins with prior research [19]. However, previous findings on healthy food store interventions [31] show that such a backlash, which some managers expected, did not occur. This difference in resistance towards plant-based versus healthy interventions may be related to the fact that being a meat-eater can be part of people’s identity [44], eliciting feelings of reduced autonomy and strong negative attitudes towards meat reduction [45]. Moreover, resistance towards choosing more plant-based options as an omnivore may be due to vegan stigmatization [46]. Attempts to promote more plant-based meat alternatives may hence elicit strong feelings of reactance among parts of the population for whom meat eating is more than just a meal (e.g., [47]). This aligns with Berke & Larson [48], who showed that framing dishes as vegetarian or vegan using labelling has a negative effect on their sales. The fear of resistance leads most managers only wanting to take small, unintrusive steps towards stimulating more plant-based purchases, such as slowly adding more plant-based products, promoting them subtly or positioning them favorably. Removing animal-based options, using pricing strategies to favor more plant-based over animal-based products, or reducing animal-based portion sizes, is not an option for most outlets. This differs from previous findings regarding stimulating healthy purchases [29], for which retailers were willing to implement pricing and portion size interventions.

Although this unwillingness to take strong action is understandable from the perspective of circumventing resistance, this is problematic from the perspective of the protein transition, as only adding more plant-based options and not disincentivizing animal-based options may be insufficient to substantially reduce its consumption. The need to do more than only adding plant-based options is supported by the increased number, and sales of, vegan options in food outlets, in combination with the relatively stable meat consumption in the Netherlands. Sales value of plant-based options has increased by 62% between 2016 and 2021 in food service [49], and by 50% between 2018 and 2020 in food retail [50], yet were only recently accompanied by a small decrease in meat consumption [13]. This decrease may even be related to the relatively large price increase of meat compared to more plant-based options due to inflation [13]. The present findings are important because they point to the difficulty of outlets intervening on price or reducing the number or portion size of animal-based options to contribute to a decrease of animal-based food consumption.

A third barrier is the lack of behavioral agency to stimulate consumption of more plant-based diets. This behavioral agency is of a different nature depending on the outlet. Agency is limited more or less objectively by a lack of autonomy on the outlet level to implement substantial changes (e.g. branch supermarket; similar to healthy interventions; [29]), by (perceived) lack of available, tasty plant-based products, or a lack of skills to prepare attractive pant-based options (similar to [24]). Feeling of agency may also be undermined by the idea that making changes on the individual outlet level is insufficient for a transition, and there is a need for policy. That the nature of difficulties with behavioural agency is diverse is important from the perspective of creating a more plant-based food environment, because it means this would require very different kinds of interventions and policies depending on the outlet. Coming back to the third research question, i.e., how managers may think they are influenced by other outlets, our results show that managers report being mostly unconcerned by what other outlets are doing. Yet, they stated that the presence of other outlets did hinder them in stimulating plant-based foods, as this could lead consumers to switch to nearby outlets, thus losing revenue.

The results also show possible facilitators for outlet managers to take action. Outlet managers are willing to move together with, or slightly faster, than demand, which they believe will increase. Thus, a motivating frame towards outlet managers could emphasize the growing demand, and the need to timely develop attractive plant-based options. Furthermore, perceived demand could be validated, by facilitating interactions between outlet managers and consumers, in which consumers articulate their actual demand. This interaction could simultaneously provide input on which specific interventions are acceptable for consumers, i.e. not invoke resistance. Indeed, Gupta et al. [29] reported engagement between consumers and food outlets to be conducive for successful implementation of healthy food store interventions.

Some individual managers are much more willing and able to take action than others. This aligns with previous research [24, 27], suggesting running a plant-based restaurant is an act of activism and is part of a restaurant owner’s self-identity. While studies on healthy interventions [29] also report managers’ individual level factors, such as knowledge, skills, and feelings of responsibility for community health as facilitators, this appears different from managers stimulating plant-based choices as part of their self-identity. Reasoning from transition theory [51], such highly motivated “front-runners” may accelerate the transition within the wider the food environment. Indeed, Ye et al. [52] suggest that emerging trends instigated by front runners can eventually lead to explosive diffusion of behavior once it takes off, but individual level inertia can greatly delay the time to take off, and it is very difficult to predict how long it will take for explosive diffusion of behavior to occur.

Research also suggests that consumers infer social norms from the food environment [53], and that social norms are considered an important determinant of consumer preferences and intentions [54, 55]. Thus, the current large assortment of animal-based foods may contribute to difficulties in behavior change toward plant-based food at the level of individual consumers, as they perceive a high norm to consume animal-based foods. Changes towards more plant-based foods in the food environment may be important to initiate a reinforcing loop, as initial interventions in the food environment may communicate the development of more positive plant-based consumption norms, subsequently leading to consumer behavior change, resulting in increased sales [56]. This in turn can change managers’ perception of demand, further facilitating changes in the food environment.

However, an important question is how changes in the food environment can be facilitated to initiate such possible mechanisms for change, in light of current perceived low consumer demand and high availability of animal-based food. One possibility is to draw on food environment policies, i.e., the fourth layer of the socio-ecological model [15]. These policies might be needed to more quickly instigate changes among a variety of food outlets [57], as also proposed by Winkler et al. [58] regarding healthy food interventions. Such policies could create a level playing field to dissuade animal-based purchases through effective means that are not attractive for food outlets to implement autonomously, by measures that affect all outlets simultaneously, such as a meat tax [59]. However, just as outlet managers fear resistance, also policymakers are hindered by feared backlash [57]. Other types of, likely less sensitive, policies could, for example, aim at increasing food outlet managers’ ability to find and prepare plant-based options, or at increasing the attractiveness of plant-based products available to them.

As part of our maximum-variation sampling we also included a butcher in the present research. Note that the butcher is different than the other outlets, considering their heavy focus on meat. At the same time, butchers are part of the food environment, and are a highly relevant type of outlet in the protein transition. Therefore, if we can better understand the drivers of butchers, and therewith facilitate realizing changes at those outlets, it could be a strong signal to (meat loving) consumers. Future research with a specific focus on butchers is needed to examine the role of butchers in the protein transition.

Finally, it is important to point out we focused on the role of food outlet managers, and we recognize that the protein transition is embedded in a complex and dynamic cultural, economic and political system, each with their own barriers. Therefore, the protein transition requires parallel intervening by policymakers, industry and health services [18] targeting different levels of the socio-ecological model [15]. For interventions in food outlets, this means that the implementation of, and response to food outlet interventions are highly context and time dependent and form dynamically within the broader system.

### Limitations

The findings from the current study should be interpreted considering three limitations. First, the way of recruiting participants has likely caused a selection bias. Second, maximum-variation sampling was used to gain input from a variety of food outlets, which means only limited conclusions about differences between types of outlets can be made. Third, the participants had different roles in their outlet, ranging from assistant-manager to managing owner. This may have influenced their responses, such as their reported autonomy to implement changes. Considering these limitations, future research should elaborate on these findings by validating results quantitatively with representative samples, by using participatory action research methodologies and by assessing the actual effect of different types of interventions on resistance and on sales.

## Conclusions

We identified three distinct types of barriers applicable to most managers, from a variety of food outlet types, for stimulating more plant-based and fewer animal-based purchases: Low perceived consumer demand, feared consumer resistance and low manager behavioral agency. These barriers are important to understand, and subsequently mitigate, as food outlet managers have an important role in the protein transition. Compared to previously identified barriers for stimulating healthy choices, the barriers ‘feared consumer resistance’ and ‘low manager behavioral agency’ seem particularly prevalent for stimulating plant-based choices. Still, a broader focus on plant-based purchases may be preferred as it contributes to not only health, but also sustainability and animal welfare. Furthermore, managers perceive a growing trend towards more plant-based demand, creating an opportunity for stimulating plant-based purchases. Currently, most potential for change seems to lie at few outlets, in which there is sufficient autonomy to make changes, and in which the manager is a particularly motivated, knowledgeable and skillful individual. Policies targeting multiple levels of the system may be needed to more quickly instigate changes, and accelerate the transition. Furthermore, interaction between managers and consumers may correct a potentially biased, low perception of demand, and lead to interventions that are acceptable for consumers, i.e., do not evoke resistance.

### Supplementary Information

Supplementary Material 1.

## Data Availability

To maintain the anonymity of the participants and their affiliated outlets, the interview transcripts are not made publicly available. The data are available for verification purposes.
