# Supplementary material for: Creating a healthy and sustainable food environment to promote plant-based food consumption: clear barriers and a gradual transition
Source: BMC Public Health. 2024 Jun 17;24:1607. doi: 10.1186/s12889-024-19121-5 (PMC11181573; doi:10.1186/s12889-024-19121-5)
Supplement: Supplementary file 1 — Supplementary Material 1. [file 12889_2024_19121_MOESM1_ESM.pdf]

Appendix: Interview protocol (translated from Dutch)

Introduction to the study (prior to the interview):

"Thank you for joining us. In this interview, I want to talk about the product range of plant-based food in relation to animal-based food at [...]. This research came about because there seems to be a trend towards eating more plant-based and less animal products, for sustainability and health reasons. Food outlets have a lot of influence on what people eat, but it is not clear how food outlets themselves view this trend, and what is actually possible and, very importantly, desirable for them. That is why we are now doing interviews with all kinds of different food outlets in Amersfoort, to gather the different perspectives.

Some questions are about how you are now, or are not, stimulating plant-based eating. I want to talk about the difference between plant-based options and animal-based options that you have in terms of product range, price, promotions and positioning. These are 4 important environmental elements that influence customer choice. I'll explain them: Product range is about how many plant-based options there are, in relation to the animal-based options. Price is about the difference in price between plant-based and animal-based options. Promotion is about what's on offer and what's being advertised. And as a 4th element: Positioning is about where a product/dish is placed in the store/on the menu. If an option is in plain sight, for example at the top of the card, or if an option is easy to grab on the counter, it is more likely to be chosen. Those 4 elements: product range, price, promotion and positioning of plant-based versus animal-based options in your... [restaurant/shop/...], that's what I want to talk about today.

The questions I'm going to ask, we'll ask all the different types of food outlets. Therefore, it is quite possible that there will be questions that, for example, would be relevant to a [...] or [...], but less, or not for you. That's no problem, we'll just quickly go through those questions.

I will first ask how this is with you now and what you may be working on. Next, I will also ask questions about what would theoretically be possible for you to be more involved in stimulating plant-based eating, if you wanted to. In other words: What is your sphere of influence; What can you influence? And what is desirable for you? This is again about the 4 elements: product range, price, promotion and positioning.

We will go through the list of 4 elements. I will ask about the differences between animal options and plant-based options for each element. I will specifically ask for vegetarian options, vegan options and finally also options with natural alternatives such as beans, nuts and legumes. I will explain these 3 categories of plant-based foods in more detail: Vegetarian is anything without meat and fish. Vegan options are vegetarian, but also without dairy and egg. Natural alternatives include beans, nuts and legumes. These are also vegan, but conversely, not all vegan options are also natural alternatives; For example, vegan fake meat burgers do not fall into this category. Perhaps there is some overlap in your answers for these 3 categories of plant-based foods. In that case, we'll just go through it quickly.

I have a list of questions that I will go through. I want to stress that there are no right or wrong answers; It's about your experience on this topic. It doesn't matter if you're into plant-based eating or why, any answer is good. Even if you don't know something, that's no problem at all, that's also an answer. And if something is not clear, please let me know, and I will explain it further.

Do you have any questions at the moment, or is something unclear?

In the consent form, you have indicated that the audio may be recorded. If that's okay with you, I'd like to start the audio recording now."

<u>Interview questions</u>:

- Could you, to begin with, tell us what your role is within [...] and what exactly does it entail?

1) Now I'll first ask a few questions about your product range of plant-based options. This is about how many plant-based options there are in relation to the number of animal-based options.

    a. How much/what is the ratio in the number of vegetarian options, vegan options, options with natural substitutes and animal options?
        i. (If necessary, ask specifically for the 3 categories):
        ii. Are the animal and plant-based options equivalent in size?
        iii. Why do you have the plant-based options you just described?

    b. Are you currently doing anything to make the ratio between the number of plant-based options and the number of animal-based options (even) more plant-based?
        i. If so, what? And why?
            1. (If necessary, ask which category it belongs to what they (want to) do: Vega, vegan, natural)
        ii. If not, would you like to? If so, what [see question above]? If not, why not?
            1. (If necessary, ask to which category the motivator/obstacle belongs: Vega, vegan, natural)

    c. Suppose, hypothetically, you would like to make the ratio between the number of plant-based options versus the number of animal-based options (even) more plant-based in 1 month, what could you do? In other words, what I would like to know is what your sphere of influence is, and where it ends.
        i. (What could you do?)
        ii. Set... [argument impediment they just mentioned] wouldn't be a problem. What could you do then?
            1. Could you just do that next week, so to speak?
        iii. What are (other) obstacles? What would make it tricky?
            1. (If necessary, ask which category it belongs to, what they could do and what obstacles they see)
                a. Does that apply to both vegetarian options , vegan options and those of options with natural substitutes? Or is there a difference between them?

    d. What would it take for you to make your offer more plant-based in 1 month, if you wanted to?
        i. Does that apply to both vegetarian options , vegan options and those of options with natural substitutes?

2) We've now talked about the product range of plant-based options. Now I want to move on to the 2nd element, which is price. This is about the possible difference in price between plant-based options and animal-based options.

    a. Is there a difference in price between vegetarian options, vegan options, options with natural substitutes, and animal-based options ? If so, how do they differ?

i. (If necessary, ask specifically for the 3 categories)
ii. What causes the difference in price?
1. Does that argument apply to both vegetarian options , vegan options and options with natural substitutes?

b. Are you currently doing anything to make the price of plant-based options lower compared to the price of animal-based options?
i. If so, what? And why?
1. (If necessary, ask which category it belongs to what they do: Vega, vegan, natural)
ii. If not, would you like to? If so, what then? Why (not)?
1. (If necessary, ask to which category the motivator/obstacle belongs: Vega, vegan, natural)

c. Suppose, hypothetically, you would like to lower the price of plant-based options in 1 month compared to the price of animal-based options, what could you do? In other words, what I would like to know is what your sphere of influence is, and where it ends.
i. (What could you do?)
ii. Imagine, ... [argument impediment they just mentioned] wouldn't be a problem. What could you do then?
1. Could you just do that next week, so to speak?
iii. What are obstacles?
1. (If necessary, ask which category it belongs to what obstacles they see: Vega, vegan, natural)

d. What would it take for you to make the price of plant-based options lower in 1 month compared to the price of animal-based options, if you wanted to?
i. (If necessary, ask which category it belongs to what they would need: Vega, vegan, natural)

3) We've talked about the price of plant-based options. Now I want to move on to the 3rd element, which is promotions. This is about what the promotion for plant-based options is like and how it might be different from the promotions for animal-based options.

a. Is there a difference in promotions for vegetarian options, vegan options , options with natural substitutes and animal options ? If so, how do they differ?
i. (If necessary, ask specifically for the 3 categories)
ii. (Why is the difference in promotions?)

b. Are you currently doing anything to make your promotions (even) more plant-based?
i. If so, what? And why?
1. (If necessary, ask which category it belongs to what they do now: Vega, vegan, of course)
ii. If not, would you like to? If so, what then? Why (not)?
1. (If necessary, ask to which category the motivator/obstacle belongs: Vega, vegan, natural)

c. Suppose, hypothetically, you wanted to make your promotions more plant-based in 1 month, what could you do? In other words, what I would like to know is what your sphere of influence is, and where it ends.
    i. (What could you do?)
    ii. Imagine, ... [argument impediment they just mentioned] wouldn't be a problem. What could you do then?
        1. Could you just do that next week, so to speak?
    iii. What are obstacles?
        1. (If necessary, ask which category it belongs to what obstacles they see: Vega, vegan, natural)

d. What would it take for you to start promoting plant-based options more in 1 month, if you wanted to?
    i. (If necessary, ask which category this applies to: Vega, vegan, natural)

4) We have now talked about the price of plant-based options. Now I want to move on to the 4th element, which is positioning. For positioning, it is important, for example, how visible an option is and how easy it is to grab. The questions are therefore about the possible difference between plant-based and animal-based options.

a. Is there a difference in positioning of vegetarian options, vegan options , options with natural substitutes and animal options ? If so, how do they differ?
    i. (If necessary, ask specifically for the 3 categories)
    ii. (Why is the difference in positioning?)

b. Are you currently doing something to position plant-based options (even) more in such a way that they are chosen more quickly?
    i. If so, what? And why?
        1. (If necessary, ask which category it belongs to what they do now: Vega, vegan, of course)
    ii. If not, would you like to? If so, what then? Why (not)?
        1. (If necessary, ask to which category the motivator/obstacle belongs: Vega, vegan, natural)

c. Suppose, hypothetically, in 1 month you wanted to position plant-based options (even) more so that they are chosen faster, what could you do? In other words, what I would like to know is what your sphere of influence is, and where it ends.
    i. (What could you do?)
    ii. Imagine, ... [argument impediment they just mentioned] wouldn't be a problem. What could you do then?
        1. Could you just do that next week, so to speak?
    iii. What are obstacles?
        1. (If necessary, ask which category it belongs to what obstacles they see: Vega, vegan, natural)

d. What would it take for you to start positioning plant-based options in 1 month so that they are chosen faster, if you wanted to?

i. (If necessary, ask which category this applies to: Vega, vegan, natural)

5) Finally, I would like to ask a question about portion sizes of animal options. Because, suppose you, as a food outlet, would like to reduce the consumption of animal products. Then you can, for example, change the offer / (the price or the promotion) of plant-based options; That's what we just talked about. But in addition, a food outlet may also be able to reduce the amount of animal content in a product, or the portion size.
   a. Is that something you're doing now, and why or why not?
   b. Is that something you would like to do?
      i. Why (not)?
   c. Is that something you could do, if you wanted to?
      i. Why (not)?
   d. What would you need to be able to do this?

6) In this interview, we talked about supply, (price, promotion, positioning) and portion sizes and what opportunities would be to encourage more plant-based eating. Now I would like to ask in an overarching way: Suppose it were possible that those elements would change and more plant-based eating would be stimulated in your business. What consequences do you think that would have? This can be both positive and negative.
   a. Then, if necessary, ask specifically:
      i. For your profit
      ii. For your impact on sustainability
      iii. For your impact on health

7) Are you influenced by other food outlets around you? If so, how?
   a. What would you do if the entrepreneurs around you suddenly started doing more with plant-based food?
   b. And what would you do if they started eating less plant-based food?

Closing of the interview

- We're almost at the end of the interview. Is there anything else I didn't ask, but would like to share, that you think is relevant to this research?
   a. I have asked about different ways in which you could or would like to have influence to get people to choose more plant-based in your ... . Is there a way I didn't ask about but there is?

- Now that we've talked quite a bit about plant-based eating and the possibilities for it at [...], I'm curious if you're more or less motivated to get started with this at the moment. Is that so, or has it not changed?
   a. If so: more or less? On a scale of 0 to 5, where 0 is no change at all and 5 is a very strong decrease/increase in motivation, how much has it changed for you?

- We would also like to take a look at the range of eateries we interview. Is it possible to get the menu/list of your offer?

- After the interviews have been analyzed, we will have a better idea of the current state of plant-based food in Amersfoort, and also where there are opportunities to make Amersfoort more plant-based. We

don't yet know what form that will take, but we want to take that knowledge back to the food outlets. It is possible to enter into dialogue with both the outlets, but also consumers and the municipality together, so all together, to learn from each other and to work together towards a desirable future. Would you possibly be interested in that, if that's the case?
